# Association of perceived job security and chronic health conditions with retirement in older UK and US workers

**DOI:** 10.1093/eurpub/ckab170

**Published:** 2022-02-01

**Authors:** Miriam Mutambudzi, Paul Flowers, Evangelia Demou

**Affiliations:** 1 Department of Public Health, Falk College, Syracuse University, Syracuse, NY, USA; 2 MRC/CSO Social and Public Health Sciences Unit, Institute of Health and Wellbeing, University of Glasgow, Glasgow, UK; 3 School of Psychological Sciences & Health, University of Strathclyde, Glasgow, UK

## Abstract

**Background:**

The relationship between job insecurity, chronic health conditions (CHCs) and retirement among older workers are likely to differ between countries that have different labor markets and health and social safety nets. To date, there are no epidemiological studies that have prospectively assessed the role of job insecurity in retirement incidence, while accounting for CHC trajectories in two countries with different welfare systems. We investigated the strength of the association between baseline job insecurity and retirement incidence over an 11-year period while accounting for CHC trajectories, among workers 50–55 years of age at baseline in the UK and USA.

**Methods:**

We performed Cox proportional hazards regression analysis, using 2006–2016 data from the Health and Retirement Study (US cohort, *n* = 570) and English Longitudinal Study on Aging (UK cohort *n* = 1052).

**Results:**

Job insecurity was associated with retirement after adjusting for CHC trajectories (HR = 0.69, 95% CI = 0.50–0.95) in the UK cohort only. CHC trajectories were associated with retirement in both cohorts; however, this association was attenuated in the US cohort, but remained significant for the medium-increasing trajectory in the UK cohort (HR = 1.41, 95% CI = 1.01–1.97) after adjustment for all covariates. Full adjustment for relevant covariates attenuated the association between job insecurity and retirement indicating that CHCs, social and health factors are contributing mechanistic factors underpinning retirement incidence.

**Conclusions:**

The observed differences in the two cohorts may be driven by macro-level factors operating latently, which may affect the work environment, health outcomes and retirement decisions uniquely in different settings.

## Introduction

Perceived job insecurity is defined as a workers perception of imminent threat to their employment status.^1^ It is a form of work-related stress that is incorporated into validated and widely used work psychosocial models such as the effort–reward imbalance and job-demand control models.^2,3^ Approximately 32% of working adults in the United States (US) report job insecurity, with a prevalence of up to 52% among some occupational sub-populations, and during economic downturns.^4,5^ In England, cohorts such as the British Birth Cohort Study 1970, Whitehall II, and the British Household Panel Survey have reported job insecurity prevalence rates between 7% and 40%.^6^ Job insecurity experienced by older adults impacts mental health and life satisfaction, is associated with poor health and health behaviors and may influence labor force participation.^6–11^

Job insecurity can impact decisions and timing of employment transitions and negatively affect worker wellbeing.^8^ Job insecurity and the potential peril of unemployment adversely impact older workers approaching retirement,^10,12^ due to concerns about re-employability, limited time to recover financially and organizational policies and practices that may discriminate against older workers.^9,10^ One study reported that there were negative and long-term effects on future employment probabilities among older adults who experienced job insecurity.^9^

Recent and chronic job insecurity is associated with an inability to meet household expenses during retirement.^13^ Research in the fields of economics and public policy indicates that the decision to stop working is substantially focused on financial preparation for retirement,^14,15^ and limited resources or financial obligations may prolong the working life.^16^ Immediate financial concerns such as ‘making ends meet’, current economy, mortgage payments, debt or loans, paying for health insurance or medical expenses are factors that influence future retirement decisions.^14^ The effects of experiencing job insecurity at one time point may therefore have lingering effects.

The decision to retire can also be influenced by personal health. Chronic health conditions (CHCs) are associated with work-related psychosocial stress and poor workability,^17^ and may impact transitions out of the workforce.^18^ Workers without CHCs are more likely to remain active workforce participants and even work beyond retirement, when compared with those with at least one chronic disease.^19,20^ CHCs are also independently associated with job insecurity, which adversely affects psychological well-being and physiological health outcomes.^21^ Job insecurity is significantly associated with increased risk of cardiovascular disease (CVD) and its risk factors, psychological distress, diabetes, and multimorbidity.^6,11^

Evidence from the literature implies that job insecurity can impact retirement decisions^14,15,22^; however, to our knowledge, these associations have not been assessed using longitudinal population data. Furthermore, CHCs are independently associated with both job insecurity and retirement^23,24^; however, it is unclear how they may impact the association between these two factors. These knowledge gaps are particularly important on an international scale.

The UK and U.S. for example, have different organizational culture, labor force participation patterns, national social security schemes and health care systems.^25–27^ Compared with the US, the UK has greater health and social welfare protections that are not tied to employment, or generating an income.^28–30^ A lack of similar safety nets in the US may make the consequences of job loss greater for American workers, in particular, if they are in poor health.^31^ These macro-level factors are likely to differentially impact job insecurity and its consequences and yield different priorities for retirement in older adults. This warrants country-level research that accounts for differences in work and social factors and potential influences of existing social safety nets.

The objective of the current study was therefore to examine the prospective associations between baseline job insecurity and incident retirement. We were further interested in assessing whether CHC trajectories moderated this relationship. Examination of this relationship in the UK and US, which have different health and social welfare programs, will provide an understanding of the potential burden of independent and co-occurring job insecurity and CHCs on retirement, and potentially indicate areas where policy may be useful in aiding transitions to retirement for older workers who are job insecure, have CHCs, or both. We hypothesized that due to the availability of different health and social welfare programs in the UK, job insecurity and CHCs would not significantly influence retirement, while the lack of similar programs in the US would result in significant associations between these factors.

## Methods

### Dataset description

We used data from the Health and Retirement Study (HRS) and the English Longitudinal Study on Aging (ELSA). Both are longitudinal cohort studies of health and retirement among American and English adults respectively, who are 50 years and older. The population sampling, content and wording of questions in these datasets were coordinated to be similar and allow for cross-national comparisons. Detailed descriptions of sampling procedures and study design for the HRS^32^ and ELSA^33^ are available elsewhere. Briefly, data for both cohorts are collected biennially, and include health, social, work-related and behavioral factors. HRS and ELSA data from 2006 to 2016 were used for the current analysis. The total sample size in 2006 was 9771 for ELSA and 18469 for HRS. Approximately 50% of the HRS sample was selected for an in-person interview. Baseline waves were selected based on initial availability of work psychosocial factors in either study. The data are de-identified and publicly available; therefore, ethical review and approval were not required by University of Glasgow.

### Inclusion/exclusion criteria

Participants were included in the study if they were (i) working for pay at baseline (*n* = 3946 ELSA, *n* = 6642 HRS); (ii) did not also indicate that they were retired (*n* = 3751 ELSA, *n* = 5861 HRS); (iii) had baseline data for the work psychosocial questionnaire, including a response to the statement ‘My job security is poor’ (*n* = 2929 ELSA, *n* = 2215 HRS); (iv) aged 50–55 years at baseline (*n* = 1221 ELSA, *n* = 594 HRS) and (v) had baseline measures plus at least one additional follow-up (*n* = 1090 ELSA, *n* = 579 HRS). The final analytic sample was 1052 ELSA and 570 HRS participants with complete data.

### Variables of interest

Self-reported retirement at each study wave was the outcome of interest. Participants were considered to be retired if they indicated that they retired and were not working for pay elsewhere. Job security was our predictor variable of interest and was measured by responses to the statement ‘My job security is poor’. Responses were measured on a four-point Likert scale (1 = Strongly disagree, 2 = Disagree, 3 = Agree, 4 = Strongly agree) and dichotomized (yes/no) for the purposes of this study. Data for job insecurity in HRS were derived from the mail-in self-administered Psychosocial Leave-Behind Questionnaire provided to participants who were selected for the in-person interview,^34^ while in ELSA, it was part of the main questionnaire administered to all participants.

An additional predictor variable of interest was CHC trajectories. We constructed the CHC trajectory variable using seven self-reported doctor-diagnosed conditions at each wave, which included diabetes, hypertension, cancer, lung disease, heart disease, stroke and arthritis. These conditions increase with age, are highly prevalent, and are associated with increased risk of subsequent adverse outcomes among older adults.^35^

Additional baseline variables of interest controlled for in the analyses included age, gender, race in HRS, partner/marital status, level of education, household income, health insurance coverage in HRS—private insurance in ELSA, current smoking status, moderate physical activity, depressive symptoms, weekly work hours, job tenure, and occupational grouping (white collar, blue collar, service work). Depressive symptoms were measured using the eight-item Center for Epidemiologic Studies Depression Scale (CESD). Exercise was defined as engaging in moderate exercise two or more times a week. To minimize missing data, in ELSA 42 participants whose education level was marked as ‘missing other’ were included as an additional category for education and labeled ‘other qualifications’.

### Statistical analysis

Baseline sample characteristics were summarized using frequencies and means. Latent class mixture models that estimated the number and size of the trajectories and assigned probability of latent membership were used to construct CHC trajectories (2006–2016).^36^ Model selection was determined using (i) Bayesian information criterion, (ii) confidence intervals (CIs), (iii) sample sizes in trajectories and (iv) average posterior probabilities above 0.7.^36^ For both cohorts, a model with linear functional form that produced three trajectory classes was the best fit for the data.

Retirement incidence over the study period is displayed using Kaplan–Meier survival curves for each CHC trajectory class. ‘Failure’ was defined as first self-report of retirement over the study period. Participants were censored if they were lost to follow-up for any reason (including death) before their retirement outcome was ascertained or if they were followed up through the last wave without retiring. The log-rank test was used to test the null hypothesis that there is no difference between job secure and insecure older adults in the probability of retirement incidence at any time point.^37^ Cox proportional hazards regression analysis was used to determine whether baseline job insecurity predicted retirement, and the role of CHC trajectories in this association. We estimated three models for each cohort. Model 1 assessed the unadjusted relationship of job insecurity and CHC trajectories, respectively, with retirement. Model 2 assessed the association between job insecurity and retirement while adjusting for CHC trajectories only. Model 3 fully adjusted for all the covariates stated above. Where our predictor variables of interest were both statistically significant in Model 3, we would further include a job insecurity × CHC trajectory interaction term.

ELSA had a small sample of non-white individuals (*n* = 37; 3.5%). Race was therefore not included in the main models to keep them comparable between the two cohorts. We however ran an additional model for the HRS cohort controlling for race (see Supplementary table S1). Additionally, approximately 47% of ELSA participants did not have a measure for job tenure. We therefore did not include this measure in the main models but ran sensitivity analysis for the participants with the measure (*n* = 553) (see Supplementary table S2). All analyses were conducted using STATA version 17.1 (StataCorp, College Station, TX, USA).

## Results

### Descriptive results

Overall, 18% of HRS and 23.76% of ELSA participants had poor job security. Mean age was 53.2 years in HRS and 52.5 years in ELSA. Total household income was higher among HRS compared with ELSA participants, while job insecure individuals in both cohorts had lower total household income relative to job secure individuals. Mean CESD scores were also higher for job insecure individuals in both cohorts. Among HRS participants with no health insurance, a larger proportion reported being job insecure (24% vs. 7%), and there was a higher proportion of job insecure blue collar and service workers in both cohorts.

Three CHC trajectory classes were identified for both cohorts; (i) no CHCs at baseline with few participants developing some conditions by the end of the study period, referred to as ‘none-low’ hereafter (referent category); (ii) low mean CHCs at baseline and increasing over time (i.e. ‘low-increasing’); and (iii) medium mean CHCs at baseline and increasing over time (i.e. ‘medium-increasing’) (table 1 and Supplementary figure S1).

In ELSA, the mean CHCs in the none-low trajectory was zero at baseline, increasing to 0.19 by 2016, with a 0–2 range of maximum CHCs. The low-increasing trajectory had a mean of 0.63 at baseline and 1.32 by 2016 (maximum CHC range = 1–3). The medium-increasing trajectory had a baseline mean of 1.64 which increased to 2.70 (maximum CHC range = 3–5). For HRS, mean number of CHCs in the none-low trajectory at baseline was 0 and 0.57 by 2016 (maximum CHC range = 0–3). The low-increasing had a mean of 0.97 at baseline and 1.69 by 2016 (maximum CHC range = 2–4). The medium-increasing trajectory had a baseline mean of 2.28 which increased to 3.59 by 2016 (maximum CHC range = 5–6).

### English longitudinal study on aging

**Figure 1:**
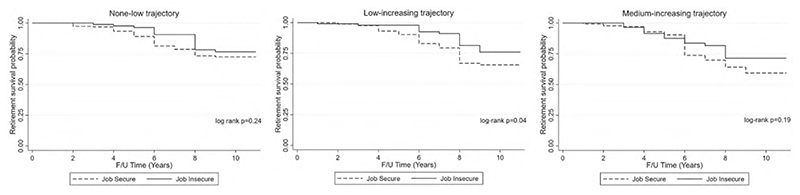
ELSA Kaplan–Meier survival curves by baseline job security status for each CHC trajectory

Within the 11-year follow-up period, there were 257 (out of 1052 working at baseline) new cases of retirement. The absolute incidence rate of retirement was higher among job secure (0.04) than job insecure employees (0.02). Kaplan–Meier curves indicated that job insecurity was not favorable for retirement and that job secure participants in the medium-increasing trajectory were more likely to retire (Figure 1). Overall, log-rank tests indicated that there were significant differences in retirement survival curves between the job secure and insecure employees in the low-increasing trajectory group only (*X* ^2^ 4.16, *P* = 0.04).

Table 2 presents the prospective associations between baseline job insecurity and CHC trajectories, and retirement over an 11-year period in ELSA and HRS participants. In Model 1, job insecurity was associated with decreased risk of retirement, and this association persisted after controlling for CHC trajectories (HR = 0.69, 95% CI = 0.50–0.95). Relative to the none-low trajectory, classification in the medium-increasing trajectory (HR = 1.51, 95% CI = 1.09–2.10) was associated with increased likelihood of retirement, while the same was not observed for the low-increasing trajectory. After full adjustment of all covariates, the association between job insecurity and retirement incidence was attenuated; however, the association between the medium-increasing trajectory and retirement persisted (HR = 1.41, 95% CI = 1.01–1.97). Retirement was also associated with education and occupational category. Relative to college graduates, those with a high school education were less likely to retire during the study period (HR = 0.71, 95% CI = 0.50–1.00). Blue collar workers were less likely to retire relative to white collar workers (HR = 0.49, 95% CI = 0.3–0.81).

### Health and retirement study

**Figure 2:**
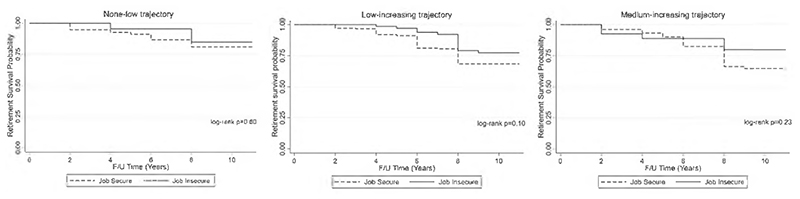
HRS Kaplan–Meier survival curves by baseline job security status for each CHC trajectory

There were 137 (out of 570 working at baseline) new cases of retirement over an 11-year period. The absolute incidence rate of retirement was higher for employees with job security (0.03) than job insecure employees (0.02). Kaplan–Meier curves indicated that job insecurity was not favorable for retirement within each of the three trajectory classes and that job secure participants in the mediumincreasing trajectory were more likely to retire; however, log-rank tests indicated that there were no significant differences in retirement survival curves for job insecure relative to job secure participants (Figure 2).

Among US workers, job insecurity was not associated with retirement, while classification in the low-increasing CHC trajectory was associated with a 55% increased likelihood of retirement (95% CI = 1.01–2.37) (table 2). After adjusting for CHC trajectories in Model 2, there remained no statistically significant association between job insecurity and retirement. Both the low-increasing (HR = 1.61, 95% CI = 1.05–2.48) and medium-increasing (HR = 1.75, 95% CI = 1.03–2.96) trajectories were however associated with increased likelihood of retirement in Model 2; however, these associations, along with job insecurity, were attenuated in Model 3 after adjustment for all covariates. Baseline age (HR = 1.22, 95% CI = 1.07–1.39) increased likelihood of retirement, while having less than a high school degree (HR = 0.33, 95% CI = 0.13–0.86) or some college reduced the likelihood of retirement (HR = 0.62, 95% CI = 0.40–0.96). Supplementary table 2 shows the association of the fully adjusted model with race included as a control variable. There were no statistically significant associations between race and retirement.

As job insecurity was not statistically significant after adjusting for all covariates in the HRS and ELSA, and CHC trajectories were also attenuated for the HRS, we did not further test for interactions in both cohorts.

## Discussion

This study assessed the temporal relationship between baseline job insecurity and retirement over an 11-year follow-up period, while accounting for CHCs in the UK and US workforces. While most studies on retirement do not take into account the impact of job insecurity,^9^ we found evidence of reduced likelihood of retirement among job insecure adults in the UK. Adjustment for covariates altered the associations, implying that the social, behavioral, health, and work factors adjusted for may partially explain the association between job insecurity and retirement incidence. Our findings also indicated that older adults with trajectories reflecting higher baseline and increasing number of CHCs over time were more likely to retire, relative to the trajectory reflecting the least number of mean CHCs. This association persisted after adjusting for all covariates in the UK cohort. The greater likelihood of retirement among those in the medium-increasing trajectory in the UK but not the US potentially implies that macro-level factors operating latently uniquely affect the work environment, health, and retirement outcomes in different settings.

Kaplan–Meier curves in our study alluded to reduced retirement among older workers who were job insecure at baseline, across all three CHC trajectory classes, while there was evidence of increased retirement among those in the trajectory reflecting greater and increasing number of CHCs over the study period. Previous studies reflect decreased retirement intentions among older workers who experience job insecurity.^13,14^ Older workers who involuntarily lose their jobs face challenges in re-employment,^9^ which may impact their current and future financial stability.^14,15^ The probability of securing re-employment in similar posts for older workers is much less than for younger workers.^38^ Among job insecure workers approaching retirement, the lack of security or consistency in employment impedes their ability to adequately plan for their financial future.^14,15^ According to the 22nd wave of the Retirement Confidence Survey, approximately a quarter of respondents were not confident they had enough money for a comfortable retirement, and over 40% felt job insecurity was a chief financial concern, with only 28% indicating they had job security.^14^ One study reported that job insecure workers expect to either continue working for pay after retirement or to delay retirement, to maintain an income and benefits such as health insurance.^13^

Our findings are supported by studies that reported an association between CHCs and retirement^18,20,23^ and are consistent with previous evidence of greater likelihood of retirement among participants with CHCs.^18,20,22^ A Dutch study reported that employees with CHCs had higher rates of retirement.^23^ Kang et al. reported significant associations between diagnosed CHCs and early ill-health retirement in Korean adults with hypertension, diabetes, lung disease, CVD, and cerebrovascular disease.^24^ Using data from Europe, another study reported that workers with CVD or diabetes had significantly increased probability of disability benefits and early retirement.^11^ Finally, two US studies found that older workers with one or more CHCs were more likely to exit the workforce.^18,20^ The complexity of managing multiple CHCs can be overwhelming and lead to anxiety, stress and decreased work ability, consequently resulting in premature exit from the workforce.^20,39^

The UK has a greater safety net, and the health and welfare protections available may in part contribute to a healthier national cohort.^29^ At baseline, 58% of ELSA (vs. 35.89% HRS) participants had no CHCs and only 10.16% (vs. 28.39% in HRS) had two or more CHCs. In the UK, universal healthcare through the National Health Service allows the population to continuously seek care for their health and wellbeing. In the US, however, variations in access and affordability of healthcare services may result in greater severity of disease,^30^ with adverse tertiary outcomes. In addition, over 55% of the US population rely on employer-based insurance,^28^ which may inadvertently encourage ill workers to remain in the workforce even as their health deteriorates.

Our study has several strengths that lend weight to our conclusions, including the use of rich prospective data from two large national cohorts. The HRS and ELSA are international sister studies, which allows for comparative research on longitudinal aging and work studies. There are, however, several limitations to this study. First, use of baseline only job insecurity did not allow us to establish causality. It is possible that over time, the factors that contribute and the degree to which they contribute to retirement decisions may vary. Second, during the 11-year follow-up, there are other factors that could have impacted the timing of retirement, including the Great Recession of 2008, as well as public, private, and personal incentives and deterrents that may not be fully captured by survey data. The Psychosocial Leave-Behind Questionnaire is administered every other wave, i.e. once every 4 years to alternating HRS subcohorts. We therefore did not have data on job insecurity for 2008 for these HRS participants. Third, we cannot rule out selection bias due to healthy worker survivor effect as workers with poorer health are more likely to exit the workforce earlier,^20,40^ and sample selection of the leave-behind questionnaire. Working participants who responded to the HRS mail in questionnaire accounted for only one-third of the mail-in respondents and may not necessarily reflect workers not selected for the in-person interview or those who chose not to complete the self-administered survey. Finally, the data do not provide additional information on whether retirement was voluntary or forced due to factors such as retrenchment.

Longitudinal assessments of the relationship between job insecurity, CHCs and retirement in the UK, the USA and beyond are scarce, and our study partially addressed this knowledge gap. Future research needs to consider longitudinal assessments of job insecurity, public and private incentives, and disincentives and how different recessions may impact different industries. An understanding of these associations at different time points in the retirement planning process and in different settings may help inform appropriate policies that are focused on building safety nets for aging workers and developing interventions on disease self-management within the workplace.

## Supplementary Material

Supplementary Material

## Figures and Tables

**Table 1:** Baseline descriptive characteristics

	HRS			ELSA	
	Job insecurity				
	No (455)	Yes (115)	No (802)	Yes (250)
	%	%	%	%
Age (mean)	53.13	53.26	52.58	52.38
Gender				
Male	40.66	49.57	43.02	48.40
Female	59.34	50.43	56.98	51.60
Education				
Other qualifications			2.37	0.80
< High school	6.37	11.30	16.71	21.60
High school	22.86	30.43	23.94	24.40
Some college	31.43	34.78	22.44	23.20
College graduate	39.34	23.48	34.54	30.00
Marital status				
Married/partner	79.34	76.52	86.66	82.00
Not married	20.66	23.48	13.34	18.00
/no partner				
Total household income (mean)	143 720.80	83 878.74	35 542.66	29 833.10
Health insurance (private health insurance in UK)				
Yes	93.41	75.65	27.43	22.00
No	6.59	24.35	72.57	78.00
Current smoker				
No	84.40	69.57	84.41	79.20
Yes	15.60	30.43	15.59	20.80
Moderate physical activity				
2+/week	68.13	65.22	74.56	72.80
> 2/week	31.87	34.78	25.44	27.20
CESD score (mean)	0.97	1.87	0.99	1.56
Work hours (mean)	41.57	40.53	35.90	35.90
Job tenure (*n*, mean)	13.04	7.90	(*n* = 421) 10.47	(*n* = 132) 8.58
Occupational category				
While collar	77.80	48.70	74.69	62.00
Service work	10.33	19.13	10.60	11.60
Blue collar	11.87	32.17	14.71	26.40
CHC trajectory class				
None-low	32.97	19.13	42.02	38.80
Low increasing	50.99	59.13	41.02	38.40
Medium-increasing	16.04	21.74	16.96	22.80

**Table 2:** Hazard ratios for the association of job insecurity and CHCs with retirement incidence in older UK and US workers

	ELSA (*n* = 1052)							HRS (*n* = 570)					
	Model 1			Model 2		Model 3		Model 1			Model 2		Model 3
	HR (95% Cl)	HR (95% Cl)		HR (95% Cl)		HR (95% Cl)		HR (95% Cl)	HR (95% Cl)		HR (95% Cl)		HR (95% Cl)
Job insecurity (ref=no)													
Yes	0.71 (0.52-0.97)			0.69 (0.5-0.95)		0.81 (0.59-1.13)		0.69 (0.44-1.09)			0.65 (0.41-1.03)		0.64 (0.39-1.05)
CHC trajectory class (ref=none-low)													
Low-increasing		1.19 (0.90-1.58)		1.19 (0.9-1.58)		1.23 (0.93-1.64)			1.55 (1.01-2.37)		1.61 (1.05-2.48)		1.38 (0.89-2.14)
Medium-increasing		1.47 (1.06-2.04)		1.51 (1.09-2.10)		1.41 (1.01-1.97)			1.67 (0.99-2.82)		1.75 (1.03-2.96)		1.44 (0.83-2.49)
Baseline age						1.38 (1.27-1.49)							1.22 (1.07-1.39)
Gender (ref = male)													
Female						1.21 (0.89-1.65)							0.90 (0.60-1.35)
Education (ref=college graduate)													
Other qualifications						0.71 (0.31-1.66)							0.33 (0.13-0.86)
< High school						0.72 (0.47-1.09)							0.80 (0.5-1.28)
High school						0.71 (0.5-1.00)							0.62 (0.4-0.96)
Some college						0.93 (0.67-1.31)							
Marital status (ref=married/partner)													0.94 (0.61-1.45)
Not married/no partner						0.88 (0.60-1.31)							1.00 (1.00-1.00)
Household income						1.00 (1.00-1.00)							
Current smoker (ref=no)													0.87 (0.53-1.43)
Yes						0.89 (0.62-1.29)							
Moderate physical activity													1.23 (0.85-1.77)
<2/week						0.92 (0.69-1.22)							1.02 (0.92-1.13)
CESD						1.01 (0.93-1.09)							
Health insurance (private in UK) (ref=yes)													
No						1.17 (0.88-1.55)							1.52 (0.81-2.85)
Work hours						0.99 (0.98-1.00)							0.98 (0.97-1.00)
Job tenure (USA only)													1.05 (1.03-1.07)
Occupational category (ref=white collar)													
Service work						0.87 (0.58-1.30)							1.66 (0.95-2.91)
Blue collar						0.49 (0.30-0.81)							1.17 (0.67-2.03)

HR: hazard ratio; Cl: confidence interval; Model 1: unadjusted; Model 2: adjusted for CHCs only; Model 3: Model 2 + age, gender, partner/marital status, level of education, health insurance, current smoker, physical activity, depressive symptoms, job tenure and occupational grouping.

## Key points

- In this paper, we show that job insecure adults have a reduced likelihood of retirement over an 11-year period.
- The association between job insecurity and retirement remains significant but is attenuated with adjustment for CHC trajectories in UK workers.
- Trajectories reflecting increasing mean CHCs are associated with increased likelihood of retirement in both cohorts.
- This association is attenuated for US workers after adjustment for health and social factors.
- The country differences observed may be potentially due to macro-level differences in social and health welfare protections.
