# Supplementary material for: Association of perceived job security and chronic health conditions with retirement in older UK and US workers
Source: Eur J Public Health. 2021 Sep 25;32(1):52–8. doi: 10.1093/eurpub/ckab170 (PMC8807079; doi:10.1093/eurpub/ckab170)
Supplement: Supplementary file 1 [file ckab170_supplementary_data.docx]

**Supplementary Material:**

Supplementary  Table S1: Hazard ratios for the association of job insecurity and CHCs with retirement incidence in older U.K workers with job tenure as a covariate (n=553)

|  | HR (95%CI) |
| --- | --- |
| Job insecurity (ref=no) |  |
| Yes | 0.78 (0.51-1.19) |
| CHC trajectory class (ref=none-low) |  |
| Low-increasing | 1.22 (0.85-1.74) |
| Medium-increasing | 1.11 (0.73-1.70) |
| Baseline age | 1.38 (1.23-1.56) |
| Gender (ref=male) |  |
| Female | 1.31 (0.87-1.96) |
| Education (ref=college graduate) |  |
| Other qualifications | 0.80 (0.33-1.95) |
| < High school | 0.79 (0.45-1.38) |
| High school | 0.74 (0.47-1.16) |
| Some college | 0.95 (0.61-1.46) |
| Marital status (ref=not married/ no partner) |  |
| No spouse/ partner | 1.25 (0.75-2.08) |
| Household income | 1.00 (1.00-1.00) |
| Current smoker (no=ref) |  |
| Yes | 0.91 (0.59-1.40) |
| Moderate physical activity |  |
| <2/week | 1.10 (0.78-1.55) |
| CESD | 0.97 (0.87-1.08) |
| Private health insurance (ref=yes) |  |
| No | 1.02 (0.71-1.48) |
| Work hours | 0.99 (0.98-1.00) |
| Occupational category (ref=white collar) |  |
| Service work | 0.80 (0.48-1.33) |
| Blue collar | 0.56 (0.29-1.06) |
| Job tenure | 1.03 (1.01-1.05) |

Supplementary  Table S2: Hazard ratios for the association of job insecurity and CHCs with retirement incidence in older U.S. workers with race as a covariate (n=570)

|  | HR (95%CI) |
| --- | --- |
| Job insecurity (ref=no) |  |
| Yes | 0.63 (0.39-1.05) |
| CHC trajectory class (ref=none-low) |  |
| Low-increasing | 1.37 (0.88-2.13) |
| Medium-increasing | 1.44 (0.82-2.51) |
| Race (ref=white) |  |
| Hispanic | 1.31 (0.66-2.58) |
| Black | 1.47 (0.82-2.65) |
| All other | 1.01 (0.37-2.81) |
| Baseline age | 1.22 (1.07-1.40) |
| Gender (ref=male) |  |
| Female | 0.88 (0.58-1.32) |
| Education (ref=college graduate) |  |
| < High school | 0.29 (0.11-0.78) |
| High school | 0.80 (0.50-1.29) |
| Some college | 0.62 (0.40-0.96) |
| Spouse/Partner (ref=married/ partner) |  |
| Not married/no partner | 0.88 (0.57-1.37) |
| Household income | 1.00 (1.00-1.00) |
| Current smoker (no=ref) |  |
| Yes | 0.88 (0.54-1.45) |
| Moderate physical activity |  |
| <2/week | 1.23 (0.85-1.78) |
| CESD | 1.02 (0.91-1.13) |
| Health insurance (ref=yes) |  |
| No | 1.57 (0.84-2.96) |
| Work hours | 0.98 (0.97-1.00) |
| Job tenure | 1.05 (1.03-1.07) |
| Occupational category (ref=white collar) |  |
| Service work | 1.63 (0.93-2.85) |
| Blue collar | 1.14 (0.65-1.98) |

Supplementary Figure S1: Trajectories of Chronic Health Conditions 2006-2016
